# Residential green space and air pollution are associated with brain activation in a social-stress paradigm

**DOI:** 10.1038/s41598-022-14659-z

**Published:** 2022-06-23

**Authors:** Annika Dimitrov-Discher, Julia Wenzel, Nadja Kabisch, Jan Hemmerling, Maxie Bunz, Jonas Schöndorf, Henrik Walter, Ilya M. Veer, Mazda Adli

**Affiliations:** 1 Department of Psychiatry and Neurosciences|CCM, Charité–Universitätsmedizin Berlin, Corporate Member of Freie Universität Berlin and Humboldt-Universität zu Berlin, Charitéplatz 1, 10117 Berlin, Germany; 2 Department of Geography, Humboldt-Universität zu Berlin, Unter den Linden 6, 10099 Berlin, Germany; 3 Department of Urban and Environmental Sociology, Helmholtz Centre of Environmental Research-UFZ, Permoserstraße 15, 04318 Leipzig, Germany; 4 Department of Environmental Hygiene, Section of Environmental Health and Health Risk Assessment, German Environment Agency, Corrensplatz 1, 14195 Berlin, Germany; 5 Department of Developmental Psychology, University of Amsterdam, Amsterdam, The Netherlands; 6 Center for Psychiatry, Psychotherapy and Psychosomatic Medicine, Fliedner Klinik Berlin, Markgrafenstrasse 34, 10117 Berlin, Germany; 7 Institute of Physical Geography and Landscape Ecology, Leibniz Universität Hannover, Schneiderberg 50, 30167 Hannover, Germany

**Keywords:** Stress and resilience, Environmental social sciences

## Abstract

We examined the influence of three major environmental variables at the place of residence as potential moderating variables for neurofunctional activation during a social-stress paradigm. Data from functional magnetic resonance imaging of 42 male participants were linked to publicly accessible governmental databases providing information on amount of green space, air pollution, and noise pollution. We hypothesized that stress-related brain activation in regions important for emotion regulation were associated positively with green space and associated negatively with air pollution and noise pollution. A higher percentage of green space was associated with stronger parietal and insular activation during stress compared with that in the control condition. More air pollution was associated with weaker activation in the same (but also extended) brain regions. These findings may serve as an important reference for future studies in the emerging field of “neuro-urbanism” and emphasize the importance of environmental factors in urban planning.

## Introduction

Urbanization is associated with several benefits, but also challenges. “City life” is, amongst others, linked to improved access to education, culture, health institutions, and employment^1^. Simultaneously, urban living comes with an increased risk of stress-related mental disorders, such as depression or anxiety disorders^2^.

Interestingly, urban living is associated with increased amygdala activity in a stress paradigm, which is a key region for emotional processing and threat detection^3,4^. Several factors could mediate this association. Apart from social factors (e.g., social density, social isolation), environmental factors (e.g., decreased exposure to nature experiences and increased exposure to air/noise pollution) have been associated with negative impacts on physiological and psychological health^5,6^.

Greater exposure to green space (GS) has been shown to be associated with improved mood, perceived general health, and increased physical activity, whereas a negative association was reported for obesity and body mass index (BMI)^7–10^. Furthermore, GS has been reported to play a part in everyday coping with stress: more GS in residential areas has been associated with a steeper decrease in the cortisol level during the day and a lower level of self-reported stress^11^. Only one functional magnetic resonance imaging (fMRI) study has investigated the association between GS and emotional wellbeing. GS exposure (as measured with ecological momentary assessments) was especially beneficial in terms of mood improvement for urban dwellers who depicted lower, possibly less regulatory activity in the dorsal prefrontal cortex during processing of aversive emotional cues. That study suggested that urban green space (UGS) exposure might be a compensating factor for reduced neuronal regulation^12^. It is important to note, that population subgroups might benefit in different ways, caused by factors like e.g. age, level of education and social support^13–15^.

Whereas GS in cities seems to promote mental wellbeing, urban air pollution has been shown to be related to neurotoxicity, neurodegeneration, worse cognitive performance, an increased risk for the recurrence of depressive symptoms, and suicide^16–21^. Across different air pollutants, particulate matter (PM) has been identified to be among the most prevalent and harmful^22^. Though less often investigated than PM, ambient gaseous nitric oxides NO_x_ and/or NO_2_ have been associated with an increased risk of dementia, cerebrovascular disease, neurodegenerative syndromes, and poorer cognitive development in children^23–25^.

A third environmental risk factor in urban settings is noise. For example, noise annoyance has been reported to lead to a chronic stress response associated with an increased release of stress hormones^26^. Several studies have indicated that noise might contribute to the development of metabolic and cardiovascular diseases, as well as to autonomic imbalance and vascular dysfunction^27–30^. Hence, GS, air pollution, and noise pollution are environmental determinants that distinguish urban areas from more rural areas, and have been shown to influence mental health: salutogenic for GS, and pathogenic for air pollution and noise pollution. A fMRI study on how air pollution and noise pollution may endanger psychological wellbeing has not been undertaken. Here, we assessed brain activation during a social-stress paradigm in male urban dwellers. Due to the menstrual cycle and hormonal fluctuations in women, stress responses have been shown to differ from men, which is why we included only men for this study^31–33^. We hypothesized that a higher percentage of residential GS and lower values of air pollution and noise pollution are associated with enhanced processing of neural stress.

## Results

### Descriptive statistics

**Figure 1:**
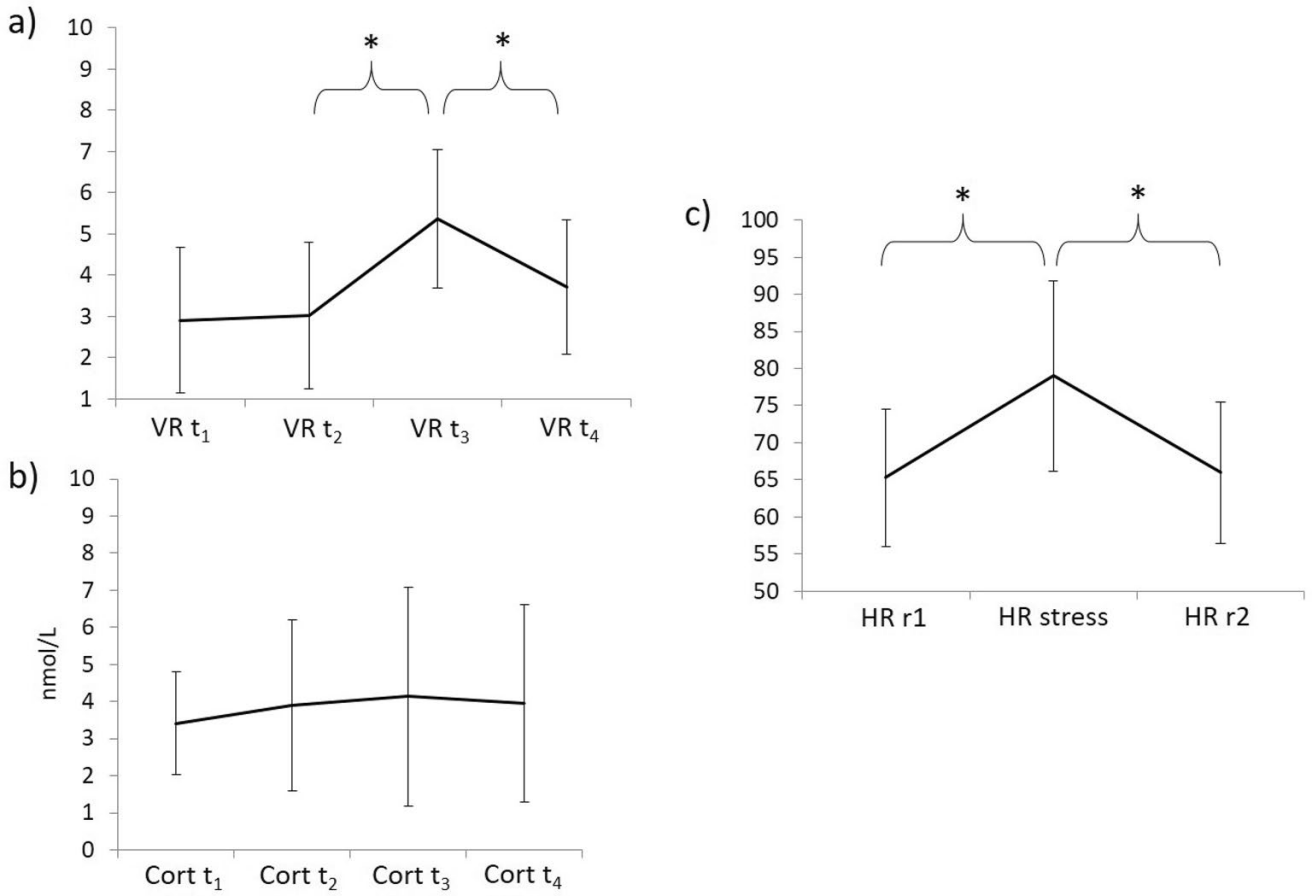
Mean (SD) for (**a**) verbal-stress rating, (**b**) cortisol concentration (in nmol/L) and (**c**) heart rate. VR = verbal-stress rating; Cort = cortisol concentration; t = time point; HR = heart rate; r = resting state; *p < 0.001.

Verbal-stress rating and cortisol concentration for t_1_ to t_4_ are depicted in Fig. 1a,b, respectively. The stress rating did not change across the four sampling time points (*p* = 0.455, *η*^2^ = 0.023), but post hoc* t*-tests demonstrated an increase from pre-stress to post-stress (*t*(41) =  − 9.346, *p* < 0.001, *d* = − 1.35) and a subsequent decrease to the next time point (*t*(41) = 7.454, *p* < 0.001, *d* = 0.989). The cortisol concentration changed over time (*F* (2.026, 74.967) = 3.158, *p* = 0.048, *η*^2^ = 0.079), but post hoc* t*-tests were not significant between any of the four sampling time points (*p* > 0.05). Heart rate changed across the three time points measured (*F* (1.129, 38.396) = 8.238, *p* = 0.005, *η*^2^ = 0.195), with a significant increase during stress (*t*(36) =  − 8.353, *p* < 0.001, *d* = − 1.116), followed by a decrease during the scan after the stress task (*t*(38) = 10.389, *p* < 0.001, *d* = 1.034) (Fig. 1c). Descriptive values are reported in Tables S1 and S2.

Descriptive statistics for the mean value of percentage GS, air pollution, and noise pollution are listed in the supplement (Tables S3 and S4). Cortisol AUCi showed a trend for an association with GS in a buffer of 5000 m (*r* =  − 0.308, *p* = 0.053). That is, the higher the amount of residential GS in a buffer of 5000 m, the lower was the cortisol AUCi in response to the social stressor. All other correlations were not significant (Table S5). When adjusted for age, the trend disappeared (*r* = − 0.184, *p* = 0.263). PM_2.5_ and PM_10_ showed negative associations with GS buffers ≥ 1500 m (− 0.74 < *r* <  − 0.47, all *p* ≤ 0.005, Bonferroni-corrected) (Table S6). For NO_2_ and NO_x_, negative correlations were found with GS buffers ≥ 1500 m (− 0.57 < *r* <  − 0.46, all *p* ≤ 0.005, Bonferroni-corrected).

### Main effect of stress according to fMRI

**Figure 2:**
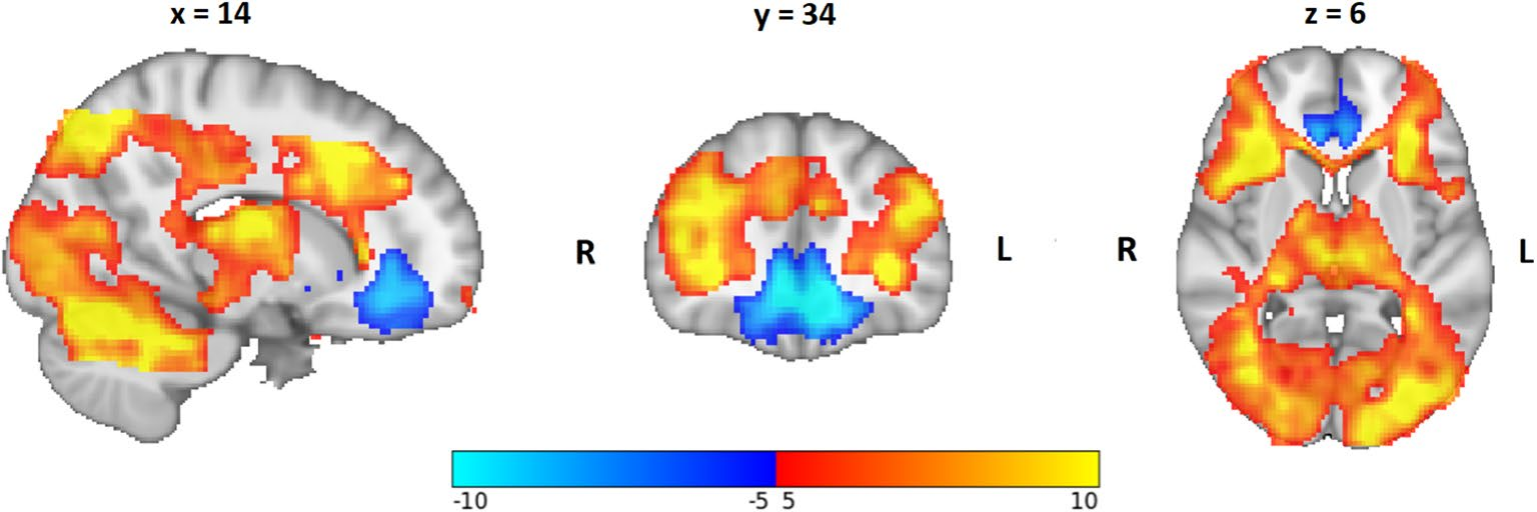
Main effect of stress > control overlaid on the 1-mm Montreal Neurological Institute (MNI) template (values represent uncorrected *t*-values). Activated brain regions are shown in red → yellow, and deactivated brain regions in blue → light-blue. R, right; L, left.

Comparison of stress with control conditions revealed stronger activity in distributed brain regions (*p* < 0.05, TFCE-corrected) (Fig. 2). These included regions central to stress processing, such as the thalamus, frontoinsular cortex, hippocampus, and amygdala. Deactivation was found in the bilateral rostral anterior cingulate cortex (rACC), posterior cingulate cortex (PCC), ventral striatum (VS), ventromedial prefrontal cortex (vmPFC), left frontal pole, and left occipital cortex (OC).

### Association between GS and brain activity

**Figure 3:**
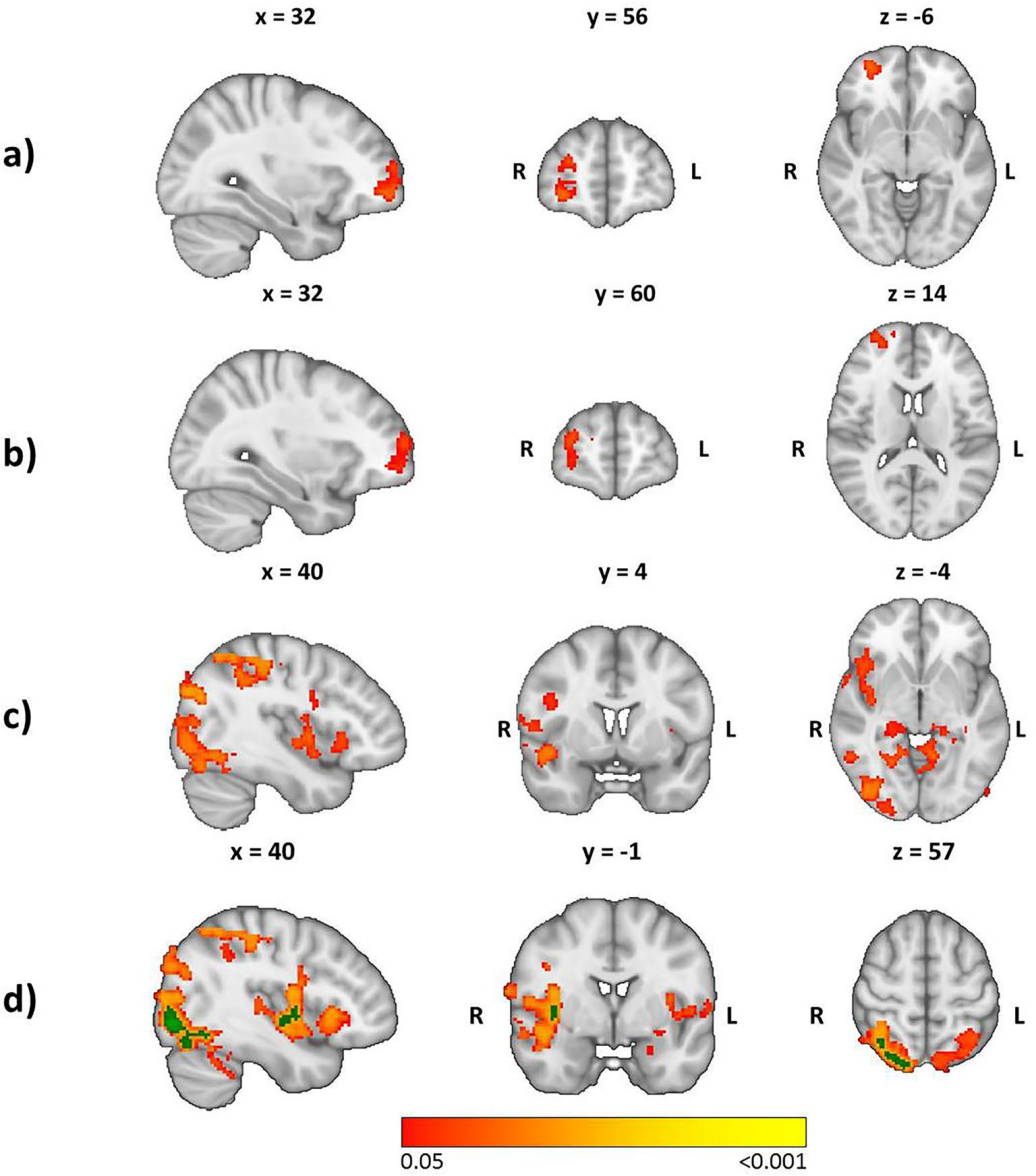
Stronger activity related to green space in a buffer of (**a**) 1500 m, (**b**) 2000 m, (**c**) 4000 m, and (**d**) 5000 m for the contrast stress > control overlaid on the 1-mm MNI template. Red-to-yellow indicates *p* < 0.05 (FWE-corrected) and green indicates *p* < 0.017 (FWE-corrected for comparison of nine buffer sizes). R, right; L, left.

GS within a buffer of 5000 m (Fig. 3d) showed an association with stress-related activation in the right insular cortex, superior parietal cortex, and lateral occipital cortex (*p* < 0.017, FWE-corrected for comparison of nine buffer sizes). These data indicated that participants with a higher percentage of GS in a buffer of 5000 m around their residence tended to have stronger activation in these regions during the stress condition compared with that in the control condition. At a more lenient FWE-corrected threshold of *p* < 0.05, additional associations appeared in the right ventromedial (vmPFC) and ventrolateral prefrontal cortex (vlPFC), and ventral striatum (VS), left and right amygdala, precuneus, ventral posterior cingulate cortex (PCC), hippocampus, and ventral anterior cingulate cortex (ACC), as well as in the left lateral occipital cortex, superior parietal cortex, fusiform gyrus and insular cortex. Using the more lenient FWE-corrected threshold, brain regions found for 4000 m (Fig. 3c) were comparable with those found for 5000 m, additionally showing associations in the left dorsolateral prefrontal cortex (dlPFC) and right fusiform cortex, whereas the buffers of 1500 m (Fig. 3a) and 2000 m (Fig. 3b) were associated positively with activity in the right vlPFC. GS within a buffer of 250 m, 500 m, 1000 m, 2500 m, and 3000 m, as well as the distance between the residence and the nearest GS ≥ 2 ha, were not associated with stress-related brain activation.

The mean time series of the significant buffers 1500 m, 2000 m, 4000 m, and 5000 m were not correlated with BMI (see Supplement Table S7 for descriptive statistics and Pearson correlation).

### Association between air pollution and brain activity

**Figure 4:**
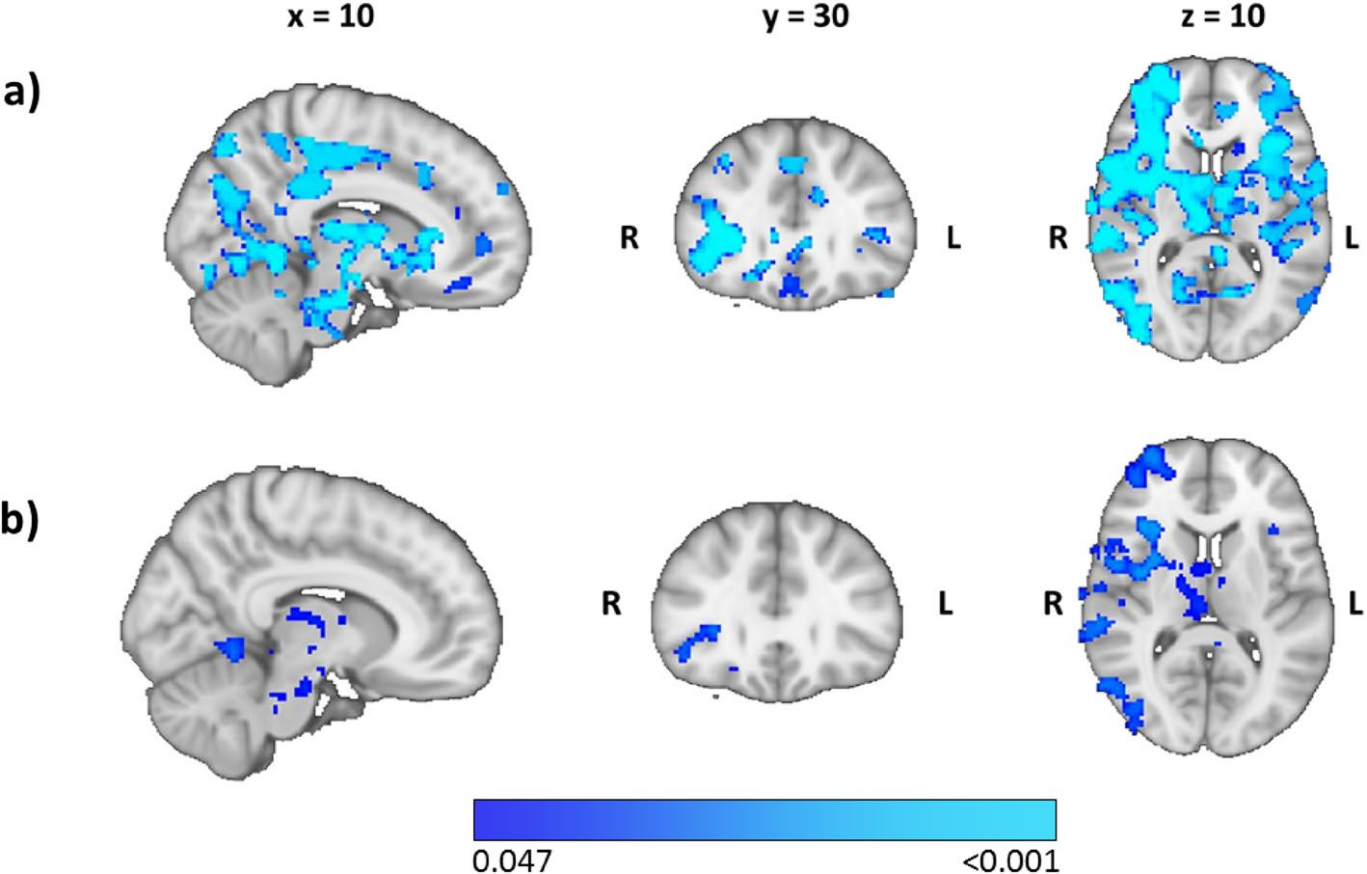
Weaker activity related to (**a**) PM_2.5_ and (**b**) PM_10_ for contrast stress > control (*p* < 0.047, corrected for multiple comparisons) overlaid on the 1-mm MNI template. R, right; L, left.

In contrast to GS, the concentration of PM_2.5_ (Fig. 4a) and PM_10_ (Fig. 4b) in residential areas showed a negative association with stress-related brain activation (*p* < 0.047, FWE-corrected for comparison of two PM values). This association was more pronounced for PM_2.5_ than for PM_10_, and comprised several regions in both hemispheres: frontoinsular cortex, hippocampus, amygdala, VS, inferior parietal cortex, thalamus, precuneus, PCC, dACC, dorsolateral PFC (dlPFC), ventrolateral PFC (vlPFC), and vmPFC. That is, participants exposed to a higher PM in their residential area had weaker stress-related activation in those brain regions. An association for the concentration of NO_2_ and NO_x_ in residential areas was not found.

### Association between noise and brain activity

An association was not found between residential noise during the day and night for a buffer of 50 m or for 100 m.

## Discussion

We wished to examine the association between urban environmental variables and brain activity during a social-stress paradigm. Successful stress induction was judged by an increase in verbal stress ratings and heart rate, as well as by a significant main effect for cortisol concentration. Overall, the analysis of the main effect of stress showed a similar activation and deactivation pattern to that stated in studies on stress processing^34^. In terms of environmental variables, we showed stronger activation in brain regions involved in emotion-based regulation of stress compared with that in the control condition for participants with higher availability of GS, particularly within a buffer of 5000 m, whereas the buffers of 1500 m, 2000 m, and 4000 m showed stronger activation at a more lenient threshold. In contrast, less activity in a more extended set of brain regions was found for participants with a higher amount of PM (especially PM_2∙5_) at their place of residence, whereas there was no association with NO or noise pollution.

For many people, nature provides an opportunity to escape the stress of daily life and to regenerate their cognitive resources^35,36^. However, until now there has been no distinct operationalization of what constitutes “restorative” GS^37^. Ekkel and de Vries stated that cumulative opportunity metrics (e.g., percentage of GS, averaged Normalized Difference Vegetation Index) show a more consistent association with health than metrics of residential proximity accessibility (e.g., distance to nearest GS, GS availability in pre-set distance)^38^. Our results showed no association between stress-related brain activation and the amount of GS for the small, “walkable” buffers of 250 m, 500 m, or 1000 m. However, we found a positive association with larger buffers, particularly 1500 m, 2000 m, and 4000 m (not corrected for the number of buffers), and 5000 m (corrected for the number of buffers), which illustrated a stronger and more widespread activity pattern with increasing buffer size. The smaller buffers of 1500 m and 2000 m were associated with stress-related activation in the right vlPFC, an area well known for cognitive control of emotional processing^39,40^. The larger buffers of 4000 m and 5000 m were associated with a more diffuse activation pattern, including the insular cortex (which is implicated in emotional perception and salience detection of cues), the vmPFC, vlPFC, dlPFC, and vACC (which are important for emotion regulation), amygdala and ventral striatum (which are related to the perception of emotional features of a stimuli), the fusiform cortex (which is important for facial emotion recognition) as well as the precuneus and PCC (which are related to self-referential thought)^34,39,41^. In general, it seems that a larger amount of GS was associated with stronger activation in brain regions that are important for regulating emotions when processing a stressful task. Surprisingly, the GS buffers of 2500 m and 3000 m did not show an association with these brain regions. However, when applying a more lenient, uncorrected threshold, we observed a similar association with activation of the right vlPFC for these buffers as that found for 1500 m and 2000 m. Interestingly, for the buffer of 3000 m, additional regions appeared to be in agreement with the regions reported for 4000 m and 5000 m, which may indicate a shift in neural associations from more rostral to dorsal stress-related brain activity with an increasing percentage of GS.

The observed associations with activity in the right vlPFC (buffers: 1500 m, 2000 m), as well as in the insular cortex, vmPFC, vlPFC, dlPFC, vACC, precuneus, and PCC (buffers: 4000 m, 5000 m) may suggest a supportive effect of GS on coping with stressful events. However, we cannot infer how the amount of GS would exert such effects. Interaction with nature becomes less likely with increasing distance for people to reach GS^42^. This matters especially for effects found within the two largest buffers. As such, our results might partly reflect an indirect association between GS and processing of neural stress, which may be influenced by the role of GS in filtering air pollution^43^. This hypothesis is supported by two results. First, we found a negative association between the amount of GS and air pollution for buffers ≥ 1500 m. Second, weaker activation was found for increased air pollution in the bilateral frontoinsular cortex, vmPFC, vlPFC, dlPFC, amygdala, hippocampus, precuneus, and PCC, which mirrored the pattern of brain regions that showed a positive association with GS. However, deactivations associated with PM comprised additional (inferior parietal cortex, thalamus, dACC) and larger areas than activations associated with green space. Interestingly, most of these regions are central nodes of three well-studied functional-connectivity networks in relation to the acute stress response: the salience network (dACC, insula, amygdala), default mode network (PCC, precuneus, hippocampus, vmPFC, inferior parietal cortex), and central executive network (dlPFC)^34^. Though fMRI research on the influence of air pollution on stress processing is still missing, a previous study employed the Trier Social Stress Test outside the scanner and reported that higher PM_2.5_ concentrations in the residing neighborhood were associated with a greater autonomic response as indicated by lower heart rate variability and higher skin conductance levels^44^. This suggests that air pollution (at least PM_2.5_) potentially inhibits an appropriate stress response, which coincides with our results in which PM may lead to a general attenuation of stress-related activity in these networks.

An association could be localized for the smaller PM_2.5_ in considerably more brain regions than for the larger PM_10_. This observation could be explained by an easier passage of the blood–brain barrier by smaller particles compared to larger ones, but also by a different chemical composition of the two particle sizes^22,45^. A previous study reported that PM_2.5_ accounted for 65% of PM_10_, suggesting reducing especially PM_2.5_ is crucial for improving air quality^46^. For the environmental variables we used, PM_2.5_ was also present in PM_10_ and, thus, may have driven the effect of PM_10_.

Surprisingly, the activity changes of the amygdala, a region which is associated with emotional processing, were opposite to what we would have expected to see, based on the assumption that green space promotes adaptive stress processing (hence we would have expected less activation of amygdala) and air pollution weakens adaptive stress processing (hence we would have expected stronger activation of amygdala)^39^. Interesting to note is that while for PM_2.5_ the significant area covered mostly anterior-lateral amygdala regions, whereas for GS only anterior parts appeared significant. The amygdala is a complex of several nuclei with different in- and output regions, which have been shown to not only correspond to negative, but also positive emotional stimuli^47,48^. However, segregating the amygdala into anatomically and functionally specific regions has been challenging and could be an interesting research question for future studies to consider^49^.

Additionally, PM has been discussed to impair the central nervous system by stimulation of pro-inflammatory cytokines and oxidative stress, which lead to neuronal loss^50,51^. Likewise, it was reported that a certain increase of PM_10_ and NO_2_ were associated with reduced volumes of many subcortical regions, amongst others, the amygdala^52^. Though volume does not equal function, air pollution-related reduction of white matter volume might play a role in the unexpected deactivation of the amygdala. Nevertheless, this and the replication of our results would have to be tested in future studies.

We did not find an association of NO_X_ or NO_2_ with stress-related activity. Studies have reported an increased risk for cerebrovascular and neurodegenerative diseases with the concentration of NO_X_ or NO_2_^23^. Furthermore, we did not find an association with noise pollution. Research has suggested that, besides objective noise pollution, subjectively perceived noise annoyance may play a part in psychological wellbeing, which was not assessed in the current study^53^.

Our study had four main limitations. First, the study cohort was relatively small, not representative, and includes only age and BMI as potential biasing factors. Thus, the results cannot be generalized to a general population or to females because stress responses differ between men and women^31^. Nor can we make assumptions for children and people with older age^54,55^. Second, the data is cross-sectional which prohibits any statement concerning causality. Third, the time of scanning and collection of environmental data did not match exactly due to the retrospective nature of our analyses: environmental data for the city of Berlin have been published only in an interval of several years. As air pollution data was extracted from the year 2015, hence 1 year before the acquisition of stress-related task activity, it could be argued that the order of events is sensible as the exposure to air pollution precedes the experiment. A different method, in terms of chronological order, had to be used for noise pollution, whose values were derived from the year 2017, a year after scanning, which might explain why we did not find effects at all. Also, it has been reported that noise exposure tasks were not successful as a stress induction method, leaving it questionable to use objective noise data for future studies^56^. Additionally, previous research suggests that besides objective noise pollution, subjectively perceived noise annoyance might play a role for psychological well-being^53^. Noise annoyance represents a conscious judgment, which is usually based on repeated experience of disturbances, an affective reaction to the disturbances or noise, and the limited ability to do something about it, which is also experienced as a loss of control^57^. And lastly, amount of green spaces was deducted from current land use data, thereby showing the least chronological conformity. As discussed above, we did find associations for rather non-walkable buffer sizes, which could have resulted from a more indirect effect in means of attenuating air pollution. Especially for green space, we have to consider the fact that we do not have information on, e.g., purpose, frequency or duration of use, which can help understand the impact of green space on health^58^. Fourth, we only had a significant main effect of salivary cortisol concentration, but post-hoc tests did not show significant differences between the time points, which might be due to the low absolute cortisol concentrations^59^. Another study reported a non-significant main effect when analyzing the entire sample due to the occurrence of non-responder^60^. Also, as we did not include a non-stressed control group, unfortunately, we cannot compare if cortisol concentrations in the stress group would have differed at all from a control group. Heart rate, however, resulted in a significant increase during the stress task and a decrease during the following non-stress resting state, which indicates a response of the cardiovascular system^61^. Likewise, verbal ratings increased from pre- to post-stress and decreased again after the second resting-state, which reflects a subjective experience of stress^62^.

Although we sought to offer an interpretation of our findings (i.e., that associations of environmental variables and brain activation are compatible with the relevance of emotion regulation during stress), our results should be interpreted as preliminary. Future studies have to find out not only whether we can replicate our findings but also if they are related to processes of emotion regulation.

## Conclusions

We found greater activation with increasing amounts of GS in brain regions relevant for regulating emotions in a stressful task. With an increasing PM concentration in the residential area, less activation in numerous regions was related to general attenuation of stress-related activity. However, we do not know whether the presence of residential GS directly influences processing of neuronal stress, whether it has indirect effects by filtering air pollutants, or whether other mediating factors are involved. This was the first study to describe changes in neuronal activity associated with the PM concentration. Depending on the place of residence, urban dwellers are exposed differently to stress-decreasing and stress-promoting environmental factors. Our results may help to understand associations of environmental inequality within a city and stress vulnerability of the brain. Further studies with larger sample sizes, equal inclusion of women and men, and preferably longitudinal observations as well as integration of different areas of expertise (e.g., “neuro-urbanism”) are needed to investigate more specifically the underlying mechanisms and implications of our findings.

## Methods

### Participants

Originally, 50 healthy male volunteers, capable of german language, were recruited via mailing lists, advertisements on a website and on flyers. Of these, four were excluded from analysis due to incidental findings in their anatomical scan (e.g., enlarged subarachnoid space, cysts) in order to have minimal distortion when matching the individual brain to a standard brain. Four more men were excluded because their addresses were outside of the city borders of Berlin. Thus, the final study cohort for analyses comprised 42 healthy men (mean age (SD) = 30.12 (5.57) years; range, 20–48 years) with a mean BMI of 23.71 (SD = 2.35; range, 19.70–30.35). All participants had a mean depression score below clinical significance (Beck Depression Inventory: mean (SD) = 4.26 (4.05); range, 0–15). 33 participants reported to drink alcohol and 23 participants reported to smoke. School education was distributed as follows: 33 participants completed a high-school diploma, six completed 10th grade, one completed a vocational baccalaureate diploma, and two completed other degrees than listed. Professional education was distributed as follows: 26 participants completed university, six completed an apprenticeship, five completed university of applied sciences, two completed other professional educations than listed and three did not complete any professional education. This experiment was part of a larger study, which took place in 2016, in which participants needed to be tested after a working day, therefore scanning always took place on a Wednesday (n = 22) or Thursday evening (n = 19) between 17:00 and 22:00. Participants refrained from taking caffeine 2 h before scanning and from strenuous physical activity for the entire day (e.g., sporting activities, fast cycling, running up stairs). Further inclusion criteria were: no shift-work, non-smoking, a late chronotype as defined by a midpoint of sleep later than 04:30 a.m. (based on Munich Chronotype Questionnaire), no current or past psychiatric disorders (based on screening from the Structured Clinical Interview for DSM-IV Axis I Disorders), no current or past physical disorders of the major organ systems^63,64^. The study protocol was approved by the Medical Ethics Committee of Charité–Universitätsmedizin Berlin (Berlin, Germany). Written informed consent was obtained from all study participants. All methods were performed in accordance with the relevant guidelines and regulations.

### Exposure variables

Urban green space (UGS) data were based on land-use data extracted from the Urban and Environment Information System provided by Berlin’s Senate Department for Urban Development and Housing for 2019^65^. Public UGS was calculated as a total sum and as a percentage of public GS with a minimum size of 0.5 ha, including urban parks, urban forests, allotment gardens, and cemeteries, in different buffer areas around street addresses. The buffers we used were 250, 500, 1000, 1500, 2000, 2500, 3000, 4000, and 5000 m. In addition, the closest distance to UGS ≥ 2 ha was calculated to indicate potential differences in accessibility of larger GS. For participants residing close to the Berlin border (n = 12), the larger buffers surpassed the land-use data of the city of Berlin. To ensure that values were not underestimated or overestimated for these participants, data from forest areas in Brandenburg (http://www.brandenburg-forst.de/LFB/client/) were additionally calculated and compared with values extracted from Berlin only by the Wilcoxon signed-rank test (see Supplement Fig. S2 and Table S3). Up to a buffer of 4000 m, no significant difference was found between the value for Berlin and Brandenburg (p > 0.05). However, the percentage for the 5000 m buffer was significantly different (*Z* = 2.201, *p* = 0.028). Therefore, analysis for this buffer was done with GS values including forest areas in Brandenburg.

Data on air quality and noise were also obtained from the Berlin’s Senate Department for Urban Development and Housing^65^. Air-quality data were provided as a modelled annual mean value for the year 2015 on a raster of 500 m × 500 m for PM_10_, PM_2.5_, NO_2_, and NO_x_. Emissions were determined at different spatial resolutions and aggregation level depending on the source group (e.g. point sources for industry and commerce, line sources for traffic (individual, rail, shipping and air traffic), heating of buildings on building block level, biogenic sources and construction sites on borough level) and aggregated to an uniform raster grid^66^. Noise data were based on a continuation of the “strategic noise maps” of Berlin created in 2017. These maps combine information on the mean noise pollution of the main sources of urban noise (e.g., road traffic, subway traffic, noise from industry and commerce, and air traffic) during the night (22:00–06:00) and day (06:00–22:00). Noise data were extracted within a buffer of 50 m and 100 m of the participant’s street address to take into account different levels of noise exposure depending on the orientation of the place of residence. For more information on the residential area studied, see Supplemental Material and Fig. S1 and S2.

### fMRI task

The ScanSTRESS task was used to induce acute social stress by carrying out figure rotations and mathematical subtraction tasks while being observed by a two-person panel who provided verbal negative feedback (incorporating elements of social-evaluative threat and unpredictability)^60^. Time pressure was introduced by an algorithm that reduced the available time to solve the set tasks depending on performance during previous trials and by presenting the remaining time via a visualized countdown (equals uncontrollability). In the case of a mistake, the comment “Incorrect!” was presented on the screen. In the case of a correct answer, but slow performance, the comment “Work faster!” was presented on the screen. During the control condition, participants merely had to match figures and numbers.

The task started after instructions from the panel with a practice run that included a control condition for figure rotation and subtraction and a stress condition for figure rotation and subtraction. Each run took 30 s, with a 10-s break in-between. After the practice run, the panel provided negative verbal feedback to increase subjective stress. Afterwards, the experimental run started with a control block consisting of 60 s of figure rotation, a 20-s break, 60 s of subtraction, a 20-s break, followed by a stress block with 60 s of figure rotation, a 20-s break, and 60 s of subtraction. This order was repeated twice so that the total duration of the task, including practice and experimental runs, spanned ~ 15 min. The adapted ScanSTRESS paradigm was programmed and presented with Presentation 18.1 provided by Neurobehavioral Systems (http://www.neurobs.com/).

**Figure 5:**
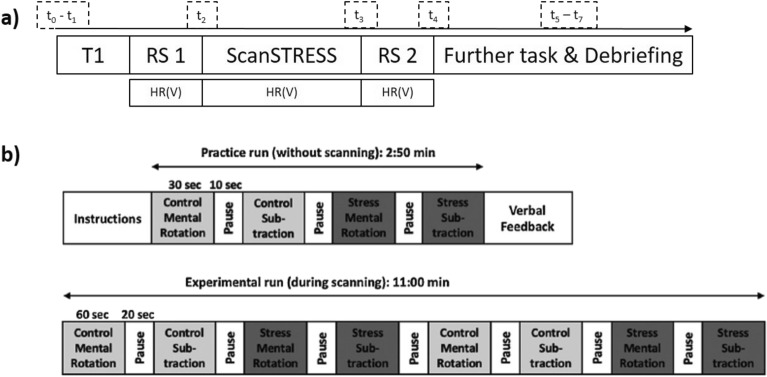
The scanning procedure (**a**) comprised a T1 MPRAGE at the start, two resting-state (RS) scans (one before and one after the stress task), followed by another functional-task scan. Saliva samples (S) were taken at eight time points: t_0_ = upon arrival, t_1_ = pre-scan, t_2_ = post resting-state 1, t_3_ = post-stress, t_4_ = post resting-state 2. Three more saliva samples were taken: after a second task (t_5_), and before (t_6_) and after (t_7_) the debriefing. HR(V) = heart rate and heart rate variability. The ScanSTRESS task (**b**) consisted of two runs inside the MRI scanner: a practice run without scanning (duration: 2:50 min) and an experimental run during scanning (duration: 11:00 min).

ScanSTRESS was embedded in a larger scanning protocol which comprised acquisition of structural scans (MPRAGE), a fieldmap, two resting state scans (pre- and post-stress) and a working-memory task with emotional distracter images (Fig. 5)^67^.

Eight saliva samples were taken throughout the entire scanning protocol: two before scanning (t_0_, t_1_), one directly before ScanSTRESS (t_2_), three after ScanSTRESS in-between other scans (t_3_, t_4_, t_5_), and two after scanning (t_6,_ t_7_). During these timepoints, participants also rated their subjective feeling of stress on a scale from 1 (“very low”) to 10 (“very high”). Heart rate was recorded during ScanSTRESS, as well as during resting-state scans preceding and following the stress task.

### Descriptive and statistical analyses

The cortisol concentration in saliva, stress rating, and heart rate were tested with a repeated-measures ANCOVA, followed by post hoc one-tailed paired-sample *t*-tests, using SPSS 25.0 (IBM, Armonk, NY, USA), including age and BMI as covariates. Effect sizes for *t*-tests were calculated using the website http://www.psychometrica.de/effect_size.html. The cortisol concentration as well as the subjective stress rating were analyzed for four time points (t_1_–t_4_). We excluded t_0_ (because it was outside the scanner environment) and t_5_ to t_7_ (because the cortisol concentration during these time points was influenced by administration of an emotional-distracter task)^67^. One participant was excluded from analyses for t_1_ and t_4_, and two participants for t_2_, due to missing values for the cortisol concentration. The cortisol area under the curve with respect to increase (AUCi) was calculated, which was used for Pearson correlations with environmental variables^68^. Two participants were excluded from the AUCi analysis due to missing values for the cortisol concentration at one or more of the sampling time points. Heart rate was not recorded for three participants during resting-state scan before the stress task (r1), for three participants during stress task and for one participant during resting-state scan after the stress task (r2), providing 37 values for the *t*-test including r1 and stress and 39 values for the *t*-test including stress and r2.

### Acquisition and processing of fMRI data

During the ScanSTRESS task, gradient-echo planar images were acquired on a 3-T scanner (Trio; Siemens, Munich, Germany) with a 32-channel head coil using the following parameters: 426 volumes; repetition time (TR) = 1560 ms; echo time (TE) = 25 ms; flip angle = 65°. Twenty-eight slices of 3-mm isotropic voxels were acquired sequentially in descending order, and auto-aligned parallel to the anterior commissure–posterior commissure line. A high-resolution T1-weighted image (magnetization prepared-rapid gradient echo; 1-mm isotropic voxels; TR = 1900 ms; TE = 2.52 ms; flip angle = 9°) and a fieldmap image (3-mm isotropic voxels; TR = 434 ms; TE = 5.19 ms; flip angle = 60°) were acquired for registration. Preprocessing was carried out using FMRIB Software Library (FSL), Advanced Normalization Tools (ANTs), and Independent Component-Analysis based Automatic Removal of Motion Artifacts (ICA-AROMA)^69–71^. Subject-level analyses were carried out in FSL using the general linear model, in which stress blocks were compared with control blocks. Group-level differences in activity between stress blocks and control blocks were assessed with a one-sample *t*-test, as well as the association of these differences with geographical data, each time including age as a covariate. Then, the resulting *t*-statistical maps underwent threshold-free cluster enhancement using the default parameter settings (H = 2, E = 0.5, C = 6), and significance testing was carried out with permutation testing (4000 iterations) using TFCE_mediation (https://github.com/trislett/TFCE_mediation)^72^. In the latter step, a null distribution of random results was generated against which empirical findings were tested. This strategy resulted in statistical images that were Family Wise Error (FEW)-corrected across the whole brain at *p* < 0.05 for the main effect of stress. Taking the mutual correlation between the nine buffer sizes of GS into account (average *r* = 0.50), the Bonferroni-corrected significance threshold was *p* = 0.017 (as calculated with SISA; http://www.quantitativeskills.com/sisa/). Taking the mutual correlation between the two PM and NO values into account (*r* = 0.92 for PM, *r* = 0.99 for NO), the Bonferroni-corrected significance threshold was *p* = 0.047 for PM and *p* = 0.05 for NO. Voxel-wise uncorrected (*t*) and corrected (TFCE *p*) statistical maps of our analyses are available on NeuroVault (http://neurovault.org/collections/9333).

In order to test possible associations of GS-related changes in brain activity with Body Mass Index (BMI), mean time series from the whole brain activity were extracted at a lenient threshold of *p* < 0.05 from significant buffers and correlated with BMI in a Pearson correlation.

More detailed information on preprocessing and analyses of fMRI data are reported in the Supplemental Material.

## Supplementary Information

Supplementary Information.

## Data Availability

Voxel-wise uncorrected (*t*) and corrected (TFCE *p*) statistical maps of our analyses are available on NeuroVault.org via this link: http://neurovault.org/collections/9333.

## References

[1] Gruebner O (2017). Cities and mental health. Dtsch. Arztebl. Int..

[2] Peen J, Schoevers RA, Beekman AT, Dekker J (2010). The current status of urban-rural differences in psychiatric disorders. Acta Psychiatr. Scand..

[3] Lederbogen F (2011). City living and urban upbringing affect neural social stress processing in humans. Nature.

[4] Pessoa L (2008). On the relationship between emotion and cognition. Nat. Rev. Neurosci..

[5] Tost H, Champagne FA, Meyer-Lindenberg A (2015). Environmental influence in the brain, human welfare and mental health. Nat. Neurosci..

[6] Adli M (2017). Neurourbanism: towards a new discipline. Lancet Psychiatry.

[7] Maas J, Verheij RA, Groenewegen PP, de Vries S, Spreeuwenberg P (2006). Green space, urbanity, and health: How strong is the relation?. J. Epidemiol. Community Health.

[8] Bratman GN, Daily GC, Levy BJ, Gross JJ (2015). The benefits of nature experience: Improved affect and cognition. Landsc. Urban Plan..

[9] De la Fuente F (2020). Green space exposure association with type 2 diabetes mellitus, physical activity, and obesity: A systematic review. Int. J. Environ. Res. Public Health.

[10] Jia P (2021). Green space access in the neighbourhood and childhood obesity. Obes. Rev..

[11] Ward Thompson C (2012). More green space is linked to less stress in deprived communities: Evidence from salivary cortisol patterns. Landsc. Urban Plan..

[12] Tost H (2019). Neural correlates of individual differences in affective benefit of real-life urban green space exposure. Nat. Neurosci..

[13] van den Berg M (2016). Visiting green space is associated with mental health and vitality: A cross-sectional study in four european cities. Health Place.

[14] Maas J, van Dillen SME, Verheij RA, Groenewegen PP (2009). Social contacts as a possible mechanism behind the relation between green space and health. Health Place.

[15] Astell-Burt T, Mitchell R, Hartig T (2014). The association between green space and mental health varies across the lifecourse. A longitudinal study. J. Epidemiol. Community Health.

[16] de Prado Bert P, Mercader EMH, Pujol J, Sunyer J, Mortamais M (2018). The effects of air pollution on the brain: A review of studies interfacing environmental epidemiology and neuroimaging. Curr. Environ. Health Rep..

[17] Lin H (2017). Exposure to air pollution and tobacco smoking and their combined effects on depression in six low- and middle-income countries. Br. J. Psychiatry J. Ment. Sci..

[18] Braithwaite I, Zhang S, Kirkbride JB, Osborn DPJ, Hayes JF (2019). Air pollution (particulate matter) exposure and associations with depression, anxiety, bipolar, psychosis and suicide risk: A systematic review and meta-analysis. Environ. Health Perspect..

[19] Liu Q (2021). Association between particulate matter air pollution and risk of depression and suicide: A systematic review and meta-analysis. Environ. Sci. Pollut. Res. Int..

[20] You R, Ho Y-S, Chang RC-C (2022). The pathogenic effects of particulate matter on neurodegeneration: A review. J. Biomed. Sci..

[21] Buoli M (2018). Is there a link between air pollution and mental disorders?. Environ. Int..

[22] Seaton A, MacNee W, Donaldson K, Godden D (1995). Particulate air pollution and acute health effects. Lancet.

[23] Migliore L, Coppede F (2009). Environmental-induced oxidative stress in neurodegenerative disorders and aging. Mutat. Res..

[24] Chang KH (2014). Increased risk of dementia in patients exposed to nitrogen dioxide and carbon monoxide: A population-based retrospective cohort study. PLoS ONE.

[25] Clark C (2012). Does traffic-related air pollution explain associations of aircraft and road traffic noise exposure on children's health and cognition? A secondary analysis of the United Kingdom sample from the RANCH project. Am. J. Epidemiol..

[26] Babisch W (2002). The noise/stress concept, risk assessment and research needs. Noise Health.

[27] Munzel T (2018). The adverse effects of environmental noise exposure on oxidative stress and cardiovascular risk. Antioxid. Redox Signal..

[28] Osborne MT (2020). A neurobiological mechanism linking transportation noise to cardiovascular disease in humans. Eur. Heart J..

[29] Osborne MT (2021). A neurobiological link between transportation noise exposure and metabolic disease in humans. Psychoneuroendocrinology.

[30] Hahad O, Prochaska JH, Daiber A, Muenzel T (2019). Environmental noise-induced effects on stress hormones, oxidative stress, and vascular dysfunction: Key factors in the relationship between cerebrocardiovascular and psychological disorders. Oxid. Med. Cell Longev..

[31] Liu JJW (2017). Sex differences in salivary cortisol reactivity to the Trier Social Stress Test (TSST): A meta-analysis. Psychoneuroendocrinology.

[32] Zandara M (2016). Acute stress and working memory: The role of sex and cognitive stress appraisal. Physiol. Behav..

[33] Kudielka BM, Hellhammer DH, Wüst S (2009). Why do we respond so differently? Reviewing determinants of human salivary cortisol responses to challenge. Psychoneuroendocrinology.

[34] van Oort J (2017). How the brain connects in response to acute stress: A review at the human brain systems level. Neurosci. Biobehav. Rev..

[35] Berto R (2014). The role of nature in coping with psycho-physiological stress: A literature review on restorativeness. Behav. Sci..

[36] Pearson DG, Craig T (2014). The great outdoors? Exploring the mental health benefits of natural environments. Front. Psychol..

[37] Taylor L, Hochuli DF (2017). Defining greenspace: Multiple uses across multiple disciplines. Landsc. Urban Plan..

[38] Ekkel ED, de Vries S (2017). Nearby green space and human health: Evaluating accessibility metrics. Landsc. Urban Plan..

[39] Etkin A, Buchel C, Gross JJ (2015). The neural bases of emotion regulation. Nat. Rev. Neurosci..

[40] Buhle JT (2014). Cognitive reappraisal of emotion: A meta-analysis of human neuroimaging studies. Cereb. Cortex.

[41] Ho TC (2016). Fusiform gyrus dysfunction is associated with perceptual processing efficiency to emotional faces in adolescent depression: A model-based approach. Front. Psychol..

[42] Coombes E, Jones AP, Hillsdon M (2010). The relationship of physical activity and overweight to objectively measured green space accessibility and use. Soc. Sci. Med..

[43] Dadvand P (2012). Surrounding greenness and exposure to air pollution during pregnancy: An analysis of personal monitoring data. Environ. Health Perspect..

[44] Miller JG, Gillette JS, Manczak EM, Kircanski K, Gotlib IH (2019). Fine particle air pollution and physiological reactivity to social stress in adolescence: The moderating role of anxiety and depression. Psychosom. Med..

[45] Calderon-Garciduenas L (2008). Long-term air pollution exposure is associated with neuroinflammation, an altered innate immune response, disruption of the blood-brain barrier, ultrafine particulate deposition, and accumulation of amyloid beta-42 and alpha-synuclein in children and young adults. Toxicol. Pathol..

[46] Zhou X (2016). Concentrations, correlations and chemical species of PM2.5/PM10 based on published data in China: Potential implications for the revised particulate standard. Chemosphere.

[47] Solano-Castiella E (2010). Diffusion tensor imaging segments the human amygdala in vivo. Neuroimage.

[48] Morrison SE, Salzman CD (2010). Re-valuing the amygdala. Curr. Opin. Neurobiol..

[49] Ball T (2009). Anatomical specificity of functional amygdala imaging of responses to stimuli with positive and negative emotional valence. J. Neurosci. Methods.

[50] Block ML, Calderon-Garciduenas L (2009). Air pollution: Mechanisms of neuroinflammation and CNS disease. Trends Neurosci..

[51] Wang Y, Xiong L, Tang M (2017). Toxicity of inhaled particulate matter on the central nervous system: neuroinflammation, neuropsychological effects and neurodegenerative disease. J. Appl. Toxicol..

[52] Cho J (2020). Long-term ambient air pollution exposures and brain imaging markers in Korean adults: The Environmental Pollution-Induced Neurological EFfects (EPINEF) Study. Environ. Health Perspect..

[53] Dümen AŞ, Şaher K (2020). Noise annoyance during COVID-19 lockdown: A research of public opinion before and during the pandemic. J. Acoust. Soc. Am..

[54] Kabisch N, Alonso L, Dadvand P, van den Bosch M (2019). Urban natural environments and motor development in early life. Environ. Res..

[55] Ranft U, Schikowski T, Sugiri D, Krutmann J, Kramer U (2009). Long-term exposure to traffic-related particulate matter impairs cognitive function in the elderly. Environ Res..

[56] Dickerson SS, Kemeny ME (2004). Acute stressors and cortisol responses: A theoretical integration and synthesis of laboratory research. Psychol. Bull..

[57] Heinecke-Schmitt R, Jäcker-Cüppers M, Schreckenberg D (2018). Reduction in the noise pollution within residential environments-what has been achieved so far?. Bundesgesundheitsblatt Gesundheitsforschung Gesundheitsschutz.

[58] Shanahan DF (2015). Toward improved public health outcomes from urban nature. Am. J. Public Health.

[59] Kudielka BM, Schommer NC, Hellhammer DH, Kirschbaum C (2004). Acute HPA axis responses, heart rate, and mood changes to psychosocial stress (TSST) in humans at different times of day. Psychoneuroendocrinology.

[60] Streit F (2014). A functional variant in the neuropeptide S receptor 1 gene moderates the influence of urban upbringing on stress processing in the amygdala. Stress.

[61] Ulrich-Lai YM, Herman JP (2009). Neural regulation of endocrine and autonomic stress responses. Nat. Rev. Neurosci..

[62] Ali N, Nitschke JP, Cooperman C, Pruessner JC (2017). Suppressing the endocrine and autonomic stress systems does not impact the emotional stress experience after psychosocial stress. Psychoneuroendocrinology.

[63] Roenneberg T, Wirz-Justice A, Merrow M (2003). Life between clocks: Daily temporal patterns of human chronotypes. J. Biol. Rhythms.

[64] Wittchen, H.-U., Zaudig, M. & Fydrich, T. *Strukturiertes Klinisches Interview für DSM-IV*. (Hogrefe, 1997).

[65] SENURBAN. *Geodata*, http://www.stadtentwicklung.berlin.de/geoinformation/fis-broker/.2014 (2019).

[66] Kindler, A., Klimeczek, H.-J. & Franck, U. In *Urban Transformations-Sustainable Urban Development Through Resource Efficiency, Quality of Life and Resilience* (eds Kabisch, S. *et al.*) 257–279 (Springer International Publishing, 2018).

[67] Dimitrov A (2021). Natural sleep loss is associated with lower mPFC activity during negative distracter processing. Cogn. Affect. Behav. Neurosci..

[68] Pruessner JC, Kirschbaum C, Meinlschmid G, Hellhammer DH (2003). Two formulas for computation of the area under the curve represent measures of total hormone concentration versus time-dependent change. Psychoneuroendocrinology.

[69] Smith SM (2004). Advances in functional and structural MR image analysis and implementation as FSL. Neuroimage.

[70] Avants BB (2011). A reproducible evaluation of ANTs similarity metric performance in brain image registration. Neuroimage.

[71] Pruim RHR (2015). ICA-AROMA: A robust ICA-based strategy for removing motion artifacts from fMRI data. Neuroimage.

[72] Lett TA (2017). Cortical surface-based threshold-free cluster enhancement and cortexwise mediation. Hum. Brain Mapp..

