# Supplementary material for: Residential green space and air pollution are associated with brain activation in a social-stress paradigm
Source: Sci Rep. 2022 Jun 23;12:10614. doi: 10.1038/s41598-022-14659-z (PMC9226020; doi:10.1038/s41598-022-14659-z)
Supplement: Supplementary file 1 — Supplementary Information. [file 41598_2022_14659_MOESM1_ESM.pdf]

Supplementary Material

Residential green space and air pollution are associated with brain activation in a social-stress paradigm

Annika Dimitrov-Discher, Julia Wenzel, Nadja Kabisch, Jan Hemmerling, Maxie Bunz, Jonas Schöndorf, Henrik Walter, Ilya M. Veer, Mazda Adli

Study location

Berlin is the most populated city in Germany. In 2018, the city had a population number of 3,748,148. Berlin is located in the lowlands of northern Germany. The administrative boundaries of the city extend >89,000 ha. Nearly 40% of the city is composed of natural areas containing 33% urban green spaces, including forest areas, public green spaces, allotment gardens and cemeteries, and 6.7% water area. These spaces are distributed heterogeneously across the city (Figure S1), with most segments of urban green space in the southwestern and southeastern parts of the city. The suburban areas close to the city border connect to large parts of urban forest, whereas other areas consist purely of agricultural land.

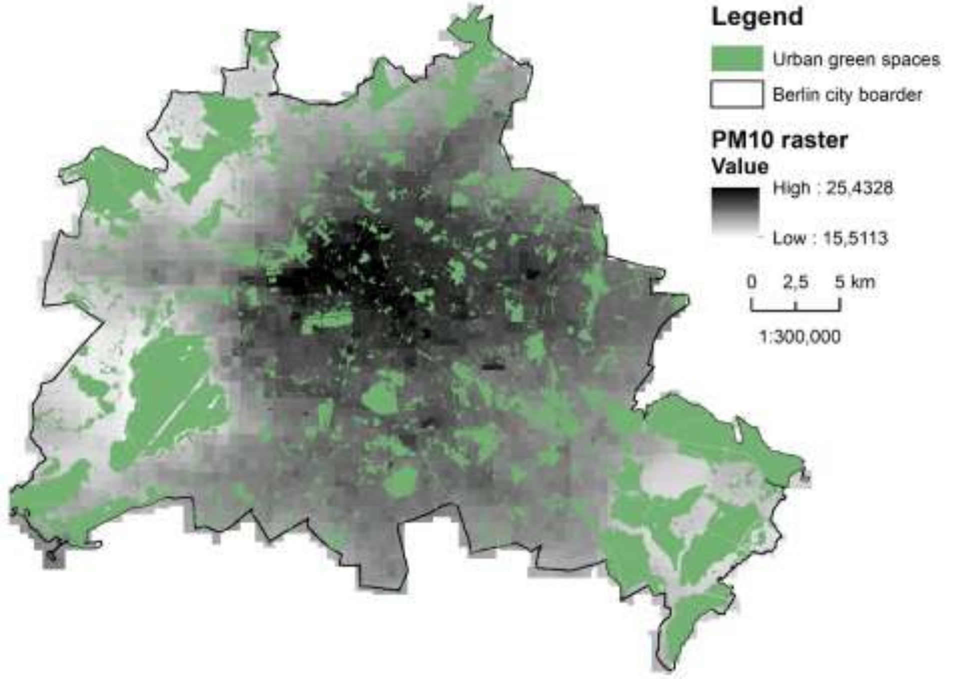

Figure S1. Distribution of urban green space in Berlin, including public green spaces, urban forests, allotment gardens and cemeteries, and PM_{10} distribution.

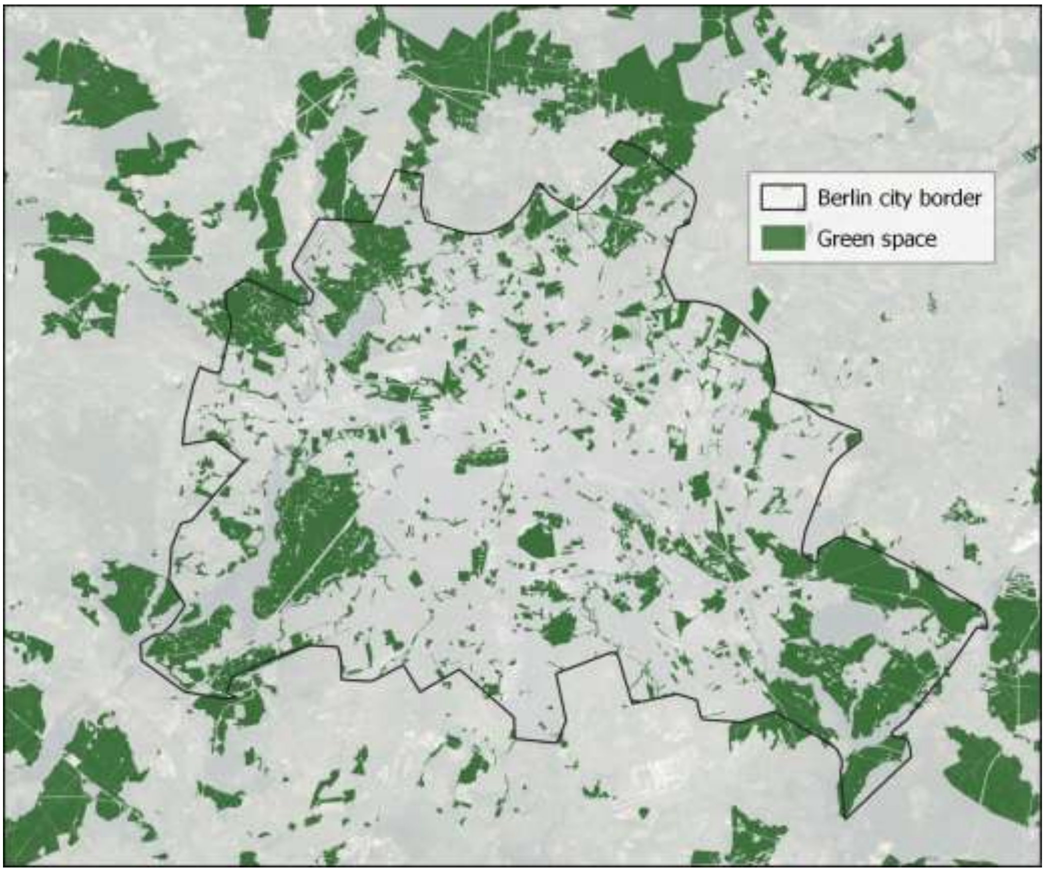

Figure S2. Distribution of urban green space in Berlin and adjacent parts of Brandenburg.

Preprocessing and analysis of fMRI data

The following preprocessing was carried out using FSL: motion correction, slice-time correction, brain extraction, and spatial smoothing with a full width at half maximum (FWHM) of 6 mm.[1] Registration parameters were obtained for the functional→structural transformation, using the fieldmap to correct for inhomogeneity artifacts (the epi_reg command within FSL employs the Boundary Based Registration [BBR] algorithm). Normalization parameters for the structural→standard space (2 mm MNI) transformation were obtained with Advanced Normalization Tools (ANTs).[2] Next, functional data were cleaned further from artifacts using ICA-AROMA, which regresses out latent signal sources (independent components) that it classifies as “noise”.[3] Lastly, a high-pass temporal filter of 420s, taking into account the period of task stimulation, was applied to the cleaned four-dimensional images, which were then normalized to standard space using the registration parameters derived previously. The slices stack was positioned so that all of the temporal lobe was included, which resulted in exclusion of dorsal parts of the brain in some participants due to the restricted number of slices that could be acquired given our TR.

First-level analyses were carried out with the fMRI Expert Analysis Tool within FSL (FEAT v6.00). A general linear model was set up and tested for each participant, including four regressors: (1) stress figure rotation (stress_fig), (2) stress subtraction (stress_maths), (3) control figure rotation (con_fig), and (4) control subtraction (con_maths). Because it was a block design, we used square as a basic waveform, with the following parameters: skip 6.24 s (4 TRs); duration of on-period = 60 s; duration until on-period returns = 280 s; pause = 25 s. All were convolved with gamma hemodynamic response function, and filtered temporally with a high-pass filter of 420s.

Table S1. Descriptive values for the cortisol concentration and verbal-stress rating

		t_1	t_2	t_3	t_4
Verbal-stress rating	*M (SD)*	2.90 (1.77)	3.02 (1.77)	5.36 (1.68)	3.71 (1.64)
	n	41	42	42	42
Cortisol concentration (nmol/L)	*M (SD)*	3.41 (1.38)	3.90 (2.30)	4.13 (2.95)	3.94 (2.66)
	n	41	40	42	41

M = mean value, *SD* = standard deviation, *n* = sample size, t = time point.

Table S2. Descriptive values for heart rate

		Resting-state 1	Stress task	Resting-state 2
Heart rate	*M (SD)*	65.26 (9.27)	78.98 (12.87)	65.99 (9.55)
	n	39	39	41

M = mean value, *SD* = standard deviation, *n* = sample size.

Table S3. Descriptive statistics and Wilcoxon signed-rank test for green space values for Berlin only and adjacent forest areas from Brandenburg.

	Berlin only *M (SD)*	Berlin + forest areas from Brandenburg *M (SD)*	Wilcoxon signed-rank test *Z* (*p*)
GS 250 m (%)	5.90 (6.51)	5.90 (6.51)	*
GS 500 m (%)	8.50 (6.66)	8.50 (6.66)	*
GS 1000 m (%)	11.26 (7.00)	11.26 (7.00)	*
GS 1500 m (%)	13.60 (9.46)	13.60 (9.46)	*
GS 2000 m (%)	15.81 (10.22)	15.81 (10.22)	*
GS 2500 m (%)	17.20 (9.96)	17.20 (9.96)	*
GS 3000 m (%)	17.80 (8.88)	17.81 (8.88)	1.342 (.180)
GS 4000 m (%)	18.63 (7.26)	18.77 (7.65)	1.826 (.068)
GS 5000 m (%)	18.33 (5.94)	18.64 (6.65)	**2.201 (.028)**

GS = green space, *M* = mean value, *SD* = standard deviation, *Z* = Wilcoxon standardized text statistic, *p* = significance value, * = identical values.

Table S4. Descriptive statistics of green space area larger than 2 ha, air pollution and noise pollution.

	M	*SD*
GS > 2 ha (m)	309.01	243.84
$PM_{2.5}$ (µg/m³)	14.18	.75
PM_{10} (µg/m³)	20.30	1.36
NO_2 (µg/m³)	19.71	2.97
NO_x (µg/m³)	28.31	5.36
Lden 50 m	56.16	8.27
Lden 100 m	55.68	7.34
Lnight 50 m	48.29	7.80
Lnight 100 m	47.85	6.87

M = mean value, *SD* = standard deviation, GS = green space, PM = particulate matter, NO = nitric oxide, L = noise.

Table S5. Correlation between AUCi and environmental determinants, without and adjusted for age.

	AUCi (*r (p)*)	
	Pearson	Partial (adjusted for age)
GS 250 m (%)	0.161 (0.321)	.160 (.332)
GS 500 m (%)	0.038 (0.815)	.071 (.669)
GS 1000 m (%)	−0.150 (0.354)	-.110 (.504)
GS 1500 m (%)	−0.139 (0.391)	-.062 (.707)
GS 2000 m (%)	−0.159 (0.327)	-.053 (.747)
GS 2500 m (%)	−0.165 (0.308)	-.048 (.773)
GS 3000 m (%)	−0.193 (0.232)	-.076 (.647)
GS 4000 m (%)	−0.271 (0.091)	-.144 (.381)
GS 5000 m (%)	**−0.30**8 (0**.053**)	-.184 (.263)
$PM_{2.5}$ (µg/m³)	0.090 (0.581)	-0.072 (0.664)
PM_{10} (µg/m³)	0.261 (0.104)	0.112 (0.499)
NO_2 (µg/m³)	0.109 (0.502)	-0.023 (0.891)
NO_x (µg/m³)	0.120 (0.459)	-0.008 (0.963)
Lden 50 m	0.024 (0.882)	-0.004 (0.982)
Lden 100 m	−0.012 (0.941)	-0.017 (0.920)
Lnight 50 m	0.050 (0.761)	0.019 (0.908)
Lden 100 m	0.012 (9.43)	0.007 (0.967)

GS = green space, PM = particulate matter, NO = nitric oxide, L = noise, *r* = correlation coefficient, *p* = significance value, Bonferroni-adjusted alpha level of $p < 0.002$ (0.05/17).

Table S6. Pearson correlation between green space and air pollution.

		GS 250 m (%)	GS 500 m (%)	GS 1000 m (%)	GS 1500 m (%)	GS 2000 m (%)	GS 2500 m (%)	GS 3000 m (%)	GS 4000 m (%)	GS 5000 m (%)
$PM_{2.5}$ (µg/m³)	*r*	0.063	0.097	−0.264	**−0.511****	**−0.606****	**−0.641****	**−0.680****	**−0.735****	**−0.736****
	p	0.693	0.542	0.091	**0.001**	**<0.001**	**<0.001**	**<0.001**	**<0.001**	**<0.001**
PM_{10} (µg/m³)	*r*	0.047	0.087	−0.239	**−0.467****	**−0.572****	**−0.611****	**−0.643****	**−0.706****	**−0.701****
	p	0.769	0.584	0.127	**0.002**	**<0.001**	**<0.001**	**<0.001**	**<0.001**	**<0.001**
NO_2 (µg/m³)	*r*	0.025	0.082	−0.330	**-0.496****	**−0.573****	**−0.568****	**−0.541****	**−0.509****	**−0.481****
	p	0.876	0.604	0.033	**0.001**	**<0.001**	**<0.001**	**<0.001**	**0.001**	**0.001**
NO_x (µg/m³)	*r*	0.024	0.083	−0.326	**−0.488****	**−0.564****	**−0.555****	**−0.524****	**−0.490****	**−0.462****
	p	0.882	0.602	0.035	**0.001**	**<0.001**	**<0.001**	**<0.001**	**0.001**	**0.002**

GS = green space, PM = particulate matter, *r* = correlation coefficient, *p* = significance value, ** Bonferroni-adjusted alpha level of $p < 0.005$ (0.05/9).

Table S7. Descriptive statistics of mean time series for significant buffers and Pearson correlation with Body Mass Index (BMI).

	Mean time series *MW (SD)*	BMI *r (p)*
GS 1500m	1.24 (1.71)	-0.048 (.761)
GS 2000m	1.33 (1.72)	-0.062 (.698)
GS 4000m	1.83 (1.35)	-0.269 (.085)
GS 5000m	1.57 (1.33)	-0.244 (.119)

GS = green space, BMI = Body Mass Index, *M* = mean value, *SD* = standard deviation, *r* = correlation coefficient, *p* = significance value.

References

1 Smith, S. M. *et al.* Advances in functional and structural MR image analysis and implementation as FSL. *NeuroImage* **23, Supplement 1**, S208-S219, doi:10.1016/j.neuroimage.2004.07.051 (2004).

2 Avants, B. B. *et al.* A reproducible evaluation of ANTs similarity metric performance in brain image registration. *Neuroimage* **54**, 2033-2044, doi:10.1016/j.neuroimage.2010.09.025 (2011).

3 Pruim, R. H. R. *et al.* ICA-AROMA: A robust ICA-based strategy for removing motion artifacts from fMRI data. *NeuroImage* **112**, 267-277, doi:10.1016/j.neuroimage.2015.02.064 (2015).
